# Rectal Injections

**Published:** 1892-11

**Authors:** 


### RECTAL INJECTIONS.

The value of flushing the colon with properly sterilized water—hot, tepid, or cold—according to the special indications present, will be readily admitted in a long list of formidable diseases, some of which may be mentioned, as follows: Habitual constipation, with or without mental depression or active cerebral manifestations due to auto-infection, being so common, might be permitted to head the list; all occult nervous affections, associated or not with the uric acid diathesis, would naturally attract our notice, as it is well known that disordered innervation is generally conducive to the absorption of poisonous products from the alimentary tract. They are also serviceable in disease dependent upon pelvic congestions, such as uterine displacements, ovarian pains, and hemorrhoids, for they contribute materially, though indirectly, to relieve the original cause, viz., hepatic insufficiency. In appendicitis, perityphlitis, and abscess, for which the *amœba coli communis* is now held responsible, they are invaluable.

Mild and even severe cases of dysentery and diarrhea are often brought to a favorable termination without enemata, but it is safe to say that, with appropriate diet, all cases would be decidedly benefited, and many fatalities avoided, by the adoption of this simple means. Enemata should find a place in the treatment of other disorders of this character, notably in the collapse of cholera morbus, yellow fever, and cholera, as well as in the conduct of cases of pleurisy or typhoid fever.—*American Therapist.*

